# A Novel Iridovirus Discovered in Deep-Sea Carnivorous Sponges

**DOI:** 10.3390/v14081595

**Published:** 2022-07-22

**Authors:** Marta Canuti, Gabrielle Large, Joost T. P. Verhoeven, Suzanne C. Dufour

**Affiliations:** Department of Biology, Memorial University of Newfoundland, 45 Arctic Ave., St. John’s, NL A1C 5S7, Canada; gjlarge@mun.ca (G.L.); verhoevenjtp@googlemail.com (J.T.P.V.)

**Keywords:** iridovirus, carnivorous sponges, Chondrocladia, Cladorhizidae, virus discovery

## Abstract

Carnivorous sponges (family Cladorhizidae) use small invertebrates as their main source of nutrients. We discovered a novel iridovirus (carnivorous sponge-associated iridovirus, CaSpA-IV) in *Chondrocladia grandis* and *Cladorhiza oxeata* specimens collected in the Arctic and Atlantic oceans at depths of 537–852 m. The sequenced viral genome (~190,000 bp) comprised 185 predicted ORFs, including those encoding 26 iridoviral core proteins, and phylogenetic analyses showed that CaSpA-IV is a close relative to members of the genus *Decapodiridovirus* and highly identical to a partially sequenced virus pathogenic to decapod shrimps. CaSpA-IV was found in various anatomical regions of six *C. grandis* (sphere, stem, root) from the Gulf of Maine and Baffin Bay and of two *C. oxeata* (sphere, secondary axis) from Baffin Bay. Partial MCP sequencing revealed a divergent virus (CaSpA-IV-2) in one *C. oxeata*. The analysis of a 10 nt long tandem repeat showed a number of repeats consistent across sub-sections of the same sponges but different between animals, suggesting the presence of different strains. As the genetic material of crustaceans, particularly from the zooplanktonic copepod order Calanoida, was identified in the investigated samples, further studies are required to elucidate whether CaSpA-IV infects the carnivorous sponges, their crustacean prey, or both.

## 1. Introduction

Sponges (phylum Porifera) are ancient metazoans characterized by their ability to use a highly efficient aquiferous system to capture food particles out of the water that is pumped through their bodies (i.e., suspension-feeding) [1]. Porifera is the most basal animal phylum, and the evolutionary and ecological success of sponges is reflected in their ability to inhabit, across a variety of depths, latitudes, and habitats, both marine and freshwater environments, where they constitute an important component of the macrobenthos [1,2,3]. Among the almost 10,000 known species [4], sponges within the family Cladorhizidae constitute an intriguing exception. Cladorhizid sponges lack or have a reduced aquiferous system and use small invertebrates as their main source of nutrients, sometimes in conjunction with suspension-feeding. Carnivorous feeding is likely an evolutionary adaption that allows sponges to survive in deep and oligotrophic environments, where cladorhizids are often found [5,6].

**Figure 1:**
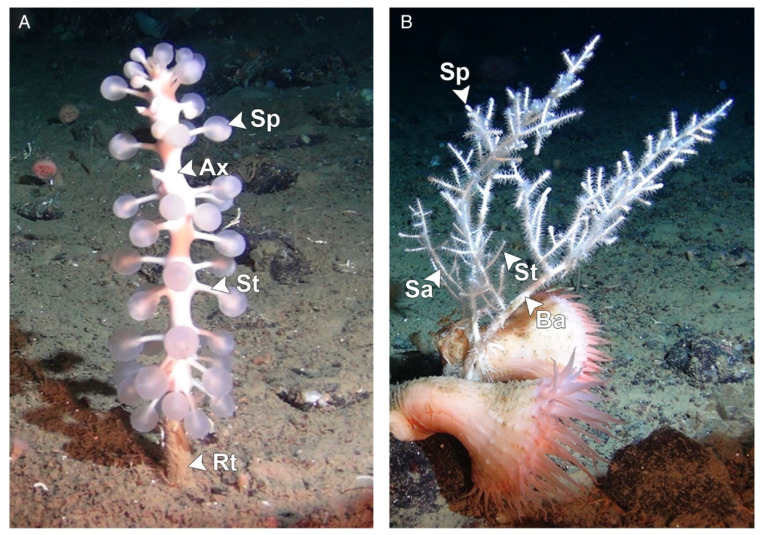
Morphology of sponges within the species *Chondrocladia grandis* (**A**) and *Cladorhiza oxeata* (**B**). Arrowheads indicate different anatomical regions. Ax: axis; Sa: secondary axis; Ba: basal axis; St: stem; Rt: root; Sp: sphere. Figure reproduced with permission from Verhoeven and Dufour 2017 [7].

Carnivorous sponges are capable of capturing and engulfing their prey, presumed to principally include small crustaceans, through a system of specialized structures and cells that include bristles, filaments, inflatable spheres, and Velcro-like spicules [5,6]. Because of this vastly different feeding strategy compared to suspension-feeding sponges, carnivorous sponges do not present a homogeneous body but possess specialized anatomical regions presumed to have different functions [6]. For example, sponges of the species *Chondrocladia grandis* consist of a single central stem (axis), from which numerous side branches (stems) terminating in inflated spheres (spheres) protrude, that terminates as a structure penetrating into the sediment (root). Other sponges, such as those of the species *Cladorhiza oxeata*, display a “bush-like” structure that consists of a larger base axis branching into thinner secondary axes that terminate in a slight swelling (sphere) (Figure 1) [7]. While previous research started to elucidate the important role of bacterial communities in enabling carnivorous feeding, no virus associated with these animals has so far been described [7,8,9,10].

During a previous study that used a metagenomic approach (ViDiT) [11] to investigate the microbiome of carnivorous sponges, we identified several genetic fragments originating from iridoviruses in *C. grandis* specimens [12]. Iridoviruses (family *Iridoviridae*) are large (120–350 nm) double-stranded DNA viruses whose icosahedral capsids, which contain a lipid membrane, may or may not be enveloped depending on how they are released from infected cells [13]. Their circularly permuted, terminally redundant dsDNA genomes vary in size between approximately 100 and 300 kbp and include between 100 and 200 open reading frames (ORFs). Consequently, their genomic map is shown as a circular molecule. Although genome structures and ORF orders vary among members of this viral family, iridoviruses are characterized by the presence of 21–26 core genes in their genome that are commonly used to determine phylogenetic relationships among members and for classification purposes [13,14,15].

Currently, the family *Iridoviridae* is divided into two phylogenetically distinct subfamilies, *Alphairidovirinae*, including three genera whose members infect primarily ectothermic vertebrates (*Lymphocystivirus*, *Megalocytivirus*, *Ranavirus*), and *Betairidovirinae*, which includes four genera whose members infect mainly invertebrates such as insects (*Chloriridovirus*, *Iridovirus*) and crustaceans (*Iridovirus*, *Daphniairidovirus*, *Decapodiridovirus*). The family now includes 22 classified viral species plus some viruses that did not yet receive official taxonomic designation [13]. Among aquatic invertebrates, iridoviruses frequently occur in crustaceans such as decapods, e.g., marine sergestid [16] or penaeid shrimps [17,18] and freshwater crayfish [18,19], and branchiopods (water fleas) [15], but they have also been observed in marine mollusks and marine annelids [20,21]. In this study, we report the identification and complete genome sequence of a novel iridovirus we identified in the carnivorous deep-sea sponges *C. grandis* and *C. oxeata*, which we named carnivorous sponge-associated iridovirus (CaSpA-IV). The aim of this study was to molecularly characterize this virus, examine its phylogenetic relationships with other known members of the *Iridoviridae*, and study its association with the carnivorous sponges in an attempt to determine its potential host(s).

## 2. Materials and Methods

### 2.1. Specimens

Sponges used in this investigation were collected in 2014 in the Gulf of Maine (East Coast of the USA at a depth of 852 m) and in 2015 in Baffin Bay (depth of 537–702 m), which is located between the west coast of Greenland and Nunavut (Canada). Overall, we investigated seven *C. grandis* from both locations and two *C. oxeata* from Baffin Bay. Specimens appeared healthy (erect, intact, with no evident biofouling, and with *Chondrocladia* spheres being inflated) on video imaging upon collection. These specimens were previously used for microbiome investigations, and further details about sample collection and preparation are available in Verhoeven et al. [7,8,11]. DNA previously isolated from several different anatomical regions of both sponge species was used in this study [7,8,11]. These included axis, stem, root, root tip, sphere, and embryo samples of *C. grandis* and base axis, secondary axis, stem, sphere, and embryo samples of *C. oxeata*. Prior to DNA isolation, sponge sections were rinsed three times with sterile nucleic acid-free PBS to remove loosely associated bacteria and other contaminants [7,8,11].

### 2.2. Full Genome Sequencing and Characterization

DNA samples from a *C. grandis* sponge from the Gulf of Maine (41°48.2302′ N, 65°41.0412′ W) collected on 27 June 2014 at 852 m of depth were used for complete viral genome sequencing. The same stem DNA sample used for the first ViDiT exploration, which was pre-treated with the NEBNext^®^ Microbiome DNA Enrichment Kit (New England Biolabs, Ipswich, MA, USA) that removes eukaryotic methylated DNA, and an un-treated DNA sample isolated from the sphere of the same sponge were used as input. High-throughput sequencing was performed at the Integrated Microbiome Resource of the Centre for Comparative Genomics and Evolutionary Bioinformatics (Dalhousie University, Halifax, NS, Canada) through Illumina sequencing after tagmentation-based library preparation [22]. All reads generated from both samples were combined together; BBDuk [23] was used to remove Illumina adapters, phiX, and low quality (<12) regions, and the remaining reads were assembled twice (default settings and custom settings with K-mer sizes of 21, 33, 55, 77) into contigs with SPAdes [24]. Contig taxonomy assignment was performed locally with BLAST using megablast (nucleotide collection database downloaded on 29 November 2020) [25] to identify iridoviral contigs.

Obtained scaffolds were connected to each other by five nested or hemi-nested PCRs followed by Sanger sequencing. Specific primers (Supplementary Table S1) were designed at both terminal ends of each scaffold, and connecting PCRs were performed with the DreamTaq Green PCR Master Mix (Thermo Fisher Scientific, Waltham, MA, USA). Amplicons were purified with AMPure XP beads (Beckman Coulter, Brea, CA, USA) and outsourced for Sanger sequencing. All sequences were investigated with Geneious R11 (Biomatters), which was also used to create the genomic map. In particular, ORFs ≥ 150 nt long were identified and annotated by hand by comparing the predicted protein with iridoviral protein sequences archived in GenBank through the BLASTP online tool (default settings at first and less stringent settings (word size: 2; gap cost existence: 9; gap cost extension: 1) for those that were unassigned during the first iteration). To assess sequence identity between viruses sequenced from the two samples and determine sequencing coverage, reads from each individual sample were mapped towards the final complete genome with Bowtie 2 [26].

### 2.3. Screening and Strain Comparisons

After the initial identification of iridovirus-like fragments with the ViDiT-CACTUS method [11,12], a nested PCR was designed to screen the various sponge samples (different anatomical regions) for CaSpA-IV. To account for possible genetic variability, primers were designed based on the partial sequences of the major capsid protein (MCP) of CaSpA-IV and its closest relative identified in GenBank (accession number EF467167). This region was chosen because of its high genetic conservation across species. Primers Irido_ScF (GGTAGGATTCGCTGGACTC) and Irido_ScR1 (ACCTGCTCGATGAGGATGTC) were used during the first amplification round (571 bp), while primers Irido_ScF and Irido_ScR2 (CAGACCTGGATGTTGGTGAG) were used during the nested amplification (447 bp). PCRs were performed with the DreamTaq Green PCR Master Mix with a final volume of 25 µL and using 1 µL DNA as input according to the following thermal profile: 5 min at 95 °C, followed by 35 (first PCR) or 25 (nested PCR) cycles of 30 s at 95 °C, 30 s at 50 °C, 35 s at 72 °C, followed by 5 min at 72 °C. Positive samples were confirmed by Sanger sequencing. Overall, 48 samples from the nine sponges were screened.

To assess sequence variability across samples, the full MCP sequence was obtained from positive samples by sequencing the products of two overlapping nested PCRs. Additionally, the genomic region between 60,003 and 60,323 nt of CaSpA-IV, including a variable number of 10 nt long sequence repeats, was obtained using primers designed to connect scaffolds A and B. PCRs were performed as delineated above but with longer extension times (1–2 min) and all used primers are available in Supplementary Table S1. Sequences were analyzed, assembled, and annotated with Geneious R11 and aligned with ClustalW [27].

### 2.4. Phylogenetic Analyses

To study the phylogenetic relationship between the discovered virus and other members of the family *Iridoviridae*, the predicted sequences of the proteins encoded by 20 core genes from CaSpA-IV were compared to those of 39 other fully sequenced iridoviruses representing all currently known iridoviral genera. Sequences were obtained by downloading the concatenated alignment file available in the resource section in the ICTV *Iridoviridae* webpage, splitting this alignment into separate single-protein alignments, and combining the sequences in these alignments with sequences of Cherax quadricarinatus iridovirus and of lizard–cricket iridovirus, previously annotated by others [19,28] and identified with BLASTP, and those of CaSpA-IV and of Daphnia iridescent virus 1 [15] identified with TBLASTX as described in Section 2.2. Details about sequences used for these analyses are available in Supplementary Table S2. An additional set of MCP sequences were used to investigate the closest relatives to CaSpA-IV and their host distributions. This included all sequenced iridoviruses not officially classified within the *Alphairidovirinae* that showed at least 50% identity over a length of at least 70% of the MCP sequence of CaSpA-IV (Supplementary Table S3). The length of these sequences was adjusted to the length of the Sergestid iridovirus, and shorter sequences were excluded.

Alignments were performed with MAFFT (E-INS-I algorithm) [29] and concatenated afterwards with Geneious R11. Phylogenetic trees were built with IQ-TREE 2 [30], using both ultrafast bootstrap approximation (ufBoot) [31] and SH-like approximate likelihood ratio test (SH-aLRT) [32] to assess branch robustness, using the best model for distance estimates between individual proteins identified as the one with the lowest Bayesian information criterion (BIC) with the ModelFinder function [33]. For the tree based on the concatenated alignment, a partition model was used [34], and different models were applied to each partition.

### 2.5. Potential Host Exploration

Contigs obtained with one of the two assemblies were examined with discontinuous megablast (nucleotide collection database downloaded on 24 May 2022) [35] to evaluate whether potential host sequences could be identified. The results were visualized with MEGAN 6 (naive algorithm; Min Score: 50; Top percent: 30) [36], potential host hits were confirmed with online BLASTN and contigs that showed high identity (at a nucleotide or protein level) to bacteria were excluded since we hypothesized that the matching sequence in GenBank could have been derived from bacteria associated to their hosts and possibly erroneously classified as eukaryotic. Similarly, contigs that showed comparable identities to animals within different subphyla were not considered. Additionally, to increase the certainty of the results, based on observations made with sequences matching sponge sequences, only hits characterized by an identity of 70% and above were considered.

## 3. Results

### 3.1. Genome Characterization of CaSpA-IV

**Figure 2:**
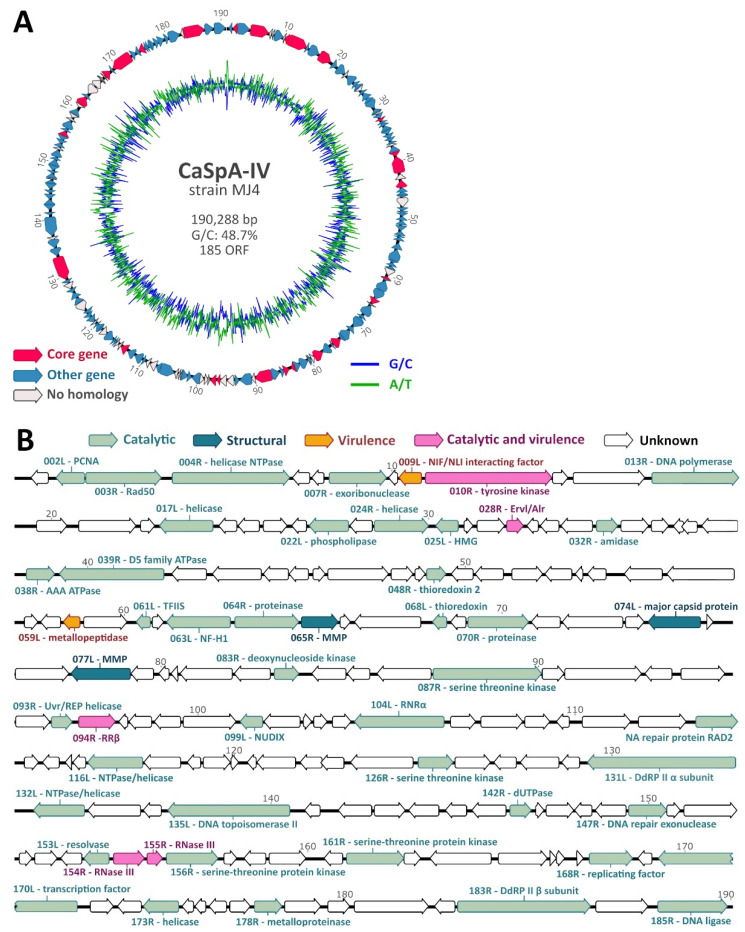
Genome organization of CaSpA-IV. Circular map of the 190,288-bp genome (**A**). The outer scale is numbered clockwise in kbp. The outer circle depicts predicted ORFs on the forward (right-pointing arrows) and reverse (left-pointing arrows) strands, while the inner circle shows the G/C (blue) and A/T (green) content throughout the genome sequence. Identified core genes are indicated in pink, while other genes are labeled depending on whether predicted proteins show homology to those of other iridoviruses, as shown by the legend. Genetic map showing predicted protein function (**B**). Arrows represent viral ORFs with their size, position, and orientation (right or left arrowheads) indicated. ORFs coding for proteins with a predicted known function are colored based on the functional category as indicated in the legend at the top. Encoded putative proteins are labeled with the sequential position in the genome followed by the ORF orientation (R: right; L: left) and predicted function. PCNA: proliferating cell nuclear antigen, Rad50: DNA double-strand break repair rad50 ATPase-like protein, HMG: high mobility group protein homolog, TFIIS: transcription elongation factor SII, NF-H1: neurofilament triplet H1-like protein, MMP: myristylated membrane protein, RNRα: ribonucleoside-diphosphate reductase large subunit, DdRP: DNA-dependent RNA polymerase. Genomic positions are expressed in Kbp.

Two samples collected from a *C. grandis* sponge from the Gulf of Maine were subjected to metagenomic DNA sequencing to obtain the complete genome of CaSpA-IV, which was previously identified in the same sponge [11,12]. After quality-polishing and trimming, the 17,021,882 sequence reads obtained assembled into several contigs, including six long iridoviral genomic fragments (three in each assembly run), which remained unconnected because of the presence of highly repetitive regions, typical of iridoviruses [13], that complicated assembly. No other contig showing homology to currently known multicellular eukaryotic viruses were identified. The two assemblies showed comparable results, and iridoviral contigs were 100% identical over their full lengths, apart from two ambiguous spots. Contigs were cut in correspondence to these ambiguous regions, and a draft genome was compiled by combining the obtained contigs. The resulting five long fragments could finally be connected by specific PCRs followed by Sanger sequencing. In the end, a circular sequence of 190,288 bp was obtained (Figure 2A). After separately mapping the sequence reads obtained from each sample to the full genome, we observed that both samples (sphere and stem from the same specimen) likely contained the same viral strain with average sequencing coverages of 16.7X (DNA from stem after methylated DNA removal) and 35X (untreated DNA from the sphere) (Supplementary Table S4). Overall, 69,451 reads mapped to the circular sequence.

The complete genome sequence contained a percentage of GC residues of approximately 49%, similar to members of the *Betairidovirinae*, which are characterized by a GC content < 50% [13]. ORF prediction resulted in 185 putative genes. Homology search based on the predicted protein sequences of these 185 genes could identify iridoviral homologs for 143 (77.3%) of them (Figure 2A). Among these, we identified all 26 core genes, which are present in almost all iridoviruses [13,14], and these were scattered across the genome (Figure 2A, Table 1). For 49 of the genes, including 25 core genes, we predicted the potential function of the encoded protein (Figure 2B). Based on sequence homology with proteins identified in other iridoviruses, these could be divided into three different functional categories, as defined by the ICTV *Iridoviridae* study group [13]: catalytic, structural (MCP and myristylated membrane proteins), and virulence.

### 3.2. Carnivorous Sponge Screening

After screening 48 samples, corresponding to various anatomical regions isolated from nine sponges, with primers designed to amplify a conserved region of the MCP, we identified 19 positive samples. In detail, all 11 samples from four *C. grandis* (four spheres, three stems, and four root samples) from the Gulf of Maine were positive, while only a few samples (8/36) collected from sponges in Baffin Bay were positive. Only two of the three *C. grandis* sponges from Baffin Bay were CaSpA-IV-positive, with the virus found in one sphere of one specimen and in four sub-sections (one stem, three root samples) of the other specimen. Finally, CaSpA-IV was found in one sphere and in one sphere and one secondary axis of the two *C. oxeata* specimens from Baffin Bay (Table 2). All partial MCP sequences except one (CaSpA-IV-2, obtained from the sphere of the *C. oxeata* specimen Bo2) were 100% identical. The 445 nt long sequence of CaSpA-IV-2 presented four nucleotide differences from all other sequences, three of which were non-synonymous.

We managed to obtain the full MCP sequence from six samples from all four *C. grandis* sponges from the Gulf of Maine, and they were all 100% identical. Unfortunately, probably due to lower DNA loads, only partial MCP sequences were obtained from Baffin Bay samples. Finally, while testing one of the connecting PCRs (fragment A to B in Supplementary Table S1), we noticed that fragments of different lengths were obtained from different samples. Therefore, an attempt was made to amplify and sequence this fragment (nt 60,003–60,323, between ORFs 060R and 061L) from all samples, but we achieved amplification for only a few of them. After sequencing PCR results, we discovered that this region contains a 10 nt long tandem repeat (TTACGAAGAC), but the number of repeats varied across samples. Interestingly, the number of repeats was consistent across sub-sections of the same sponges but different between investigated individuals, and we found two instances in which samples from the Gulf of Maine and those from Baffin Bay contained viruses with the same number of repeats (Table 2). While the sequenced fragments represented the prevailing virus strain, we cannot exclude that fragments with a different number of repeats were present in those samples but at a lower concentration.

### 3.3. Phylogenetic Relationships between CaSpA-IV and Other Iridoviruses

**Figure 3:**
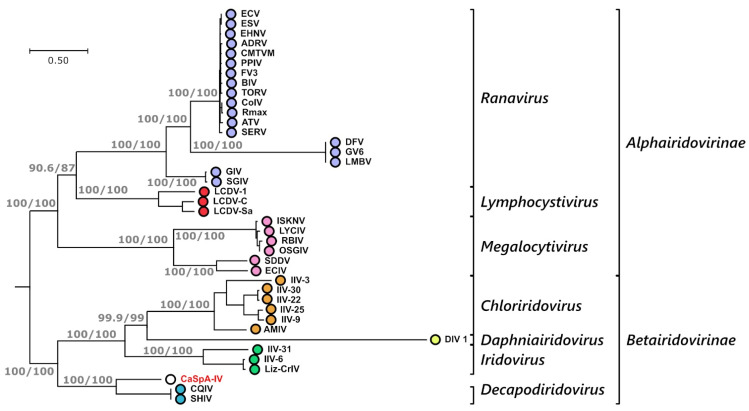
Phylogenetic analysis of CaSpA-IV and other iridoviruses. The maximum likelihood phylogenetic tree was built using IQ-Tree and a partition model with a concatenation of 20 alignments of predicted protein sequences encoded by the core genes common to all iridoviruses (Table 1). Trees built with the individual alignments and the respective distance models used for phylogenetic inference are available in Supplementary Figure S1. The outcomes of the SH-aLRT and bootstrap test (1000 replicates) are shown for the main nodes. Subfamily and genus designations are indicated on the right, and the virus identified in this study is indicated in red. Virus full names and accession numbers are available in Supplementary Table S2.

To evaluate the phylogenetic relationships between CaSpA-IV and fully sequenced viruses within the family *Iridoviridae*, we built a phylogenetic tree using a concatenated alignment of proteins predicted from the 20 core genes we could identify in all iridoviruses (Table 1). In the resulting tree, the distinction between the two viral sub-families, as well as among all known genera, was very clear, and each of these groupings was highly supported (Figure 3). In this analysis, CaSpA-IV clustered within a clade including the two decapodiridoviruses Shrimp hemocyte iridescent virus (SHIV), sequenced from the marine prawn *Litopenaeus vannamei* [37], and Cherax quadricarinatus iridovirus (CQIV), infecting the homonymous freshwater crayfish [19]. This clade occupied an intermediate position between alphairidoviruses and other betairidoviruses.

Over a concatenated alignment of the 25 shared core proteins (Table 1), CaSpA-IV was 51.2% identical to QCIV and 48.5% identical to SHIV. Differently from these two decapodiridoviruses, CaSpA-IV possessed all 26 core genes, including the one for the small subunit of the ribonucleotide reductase, which is absent from SHIV and QCIV. In most of the 20 trees built with individual proteins (Supplementary Figure S1), CaSpA-IV clustered with these two viruses, with the noticeable exceptions of the three identified structural proteins: the two myristylated membrane proteins (MMP, ORFs 065R and 077L) and the MCP (ORF 074L). In these three cases, CaSpA-IV sequences formed a single-branch clade, and the lack of close relatedness between CaSpA-IV and the two decapodiridoviruses was also confirmed by the sequence identity analyses (Table 1).

**Figure 4:**
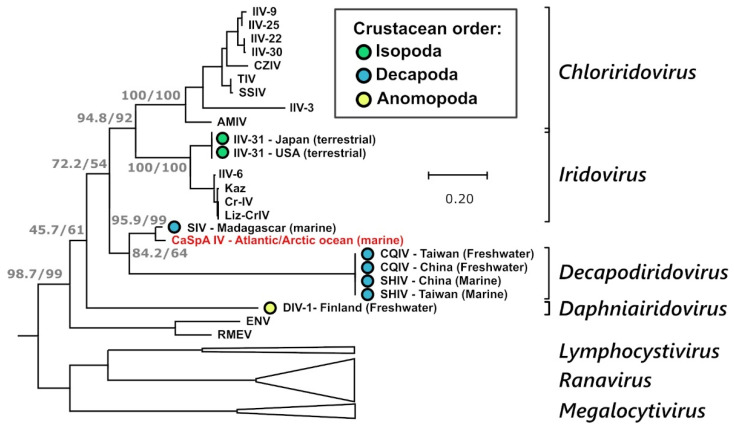
Phylogenetic analysis of the MCP of CaSpA-IV. The maximum likelihood phylogenetic tree was built with a 341 aa alignment of the MCP protein using IQ-Tree with the LG + I + G4 model. The outcomes of the SH-aLRT and bootstrap test (1000 replicates) are shown for the main nodes. Branches corresponding to genera of the *Alphairidovirinae* are collapsed and are indicated by white triangles. Genus designations, when available, are indicated on the right, and the virus identified in this study is indicated in red. Viruses of crustaceans are labeled with a colored circle as indicated in the legend, and the country of identification and the environment where the crustacean hosts were sampled are indicated in the strain name. Virus full names and accession numbers are available in Supplementary Table S3.

Since the MCP, which is highly conserved among iridoviruses, is often used for virus identification and phylogenetic analyses [13], a higher number of MCP sequences are available for comparison. Therefore, we built an additional phylogenetic tree using only MCP sequences and including also partially sequenced viruses (Figure 4). In this tree, it is clearly visible that CaSpA-IV’s closest relative is the Sergestid iridovirus (SIV), a virus causing high mortality and blue–green opalescence in the decapod shrimp species *Acetes erythraeus* [16]. In this figure, CaSpA-IV clusters with all known viruses of decapods, although the clade is poorly supported. Interestingly, the genetic distance between the subclade CaSpA-IV/SIV and the two decapodiridoviruses CQIV and SHIV was much greater than that between viruses belonging to one genus in other clades. Nonetheless, while betairidoviruses were identified several times in different crustacean orders (Figure 4), we could not find any other MCP sequence recovered from members of the Porifera (Supplementary Table S3). Interestingly, over the partially sequenced MCP, CaSpA-IV-2 was more divergent from SIV than CaSpA-IV (Supplementary Figure S2).

Interestingly, several predicted CaSpA-IV proteins showed homology with proteins annotated in scaffolds obtained through marine sample metagenomic sequencing. Particularly, these included several contigs from the iridovirus LCIVAC01 identified in deep-sea sediments of the Arctic Ocean (depth of 3236 m, 1 m below the sea floor) [38], two contigs sequenced from the giant virus fraction (0.22–1.6 um) of Mediterranean Sea water samples collected at a depth of 5–70 m [39], and seven contigs sequenced from sediment samples from the Bothnian Sea [40] (between Finland and Sweden at a depth of 33 m) (Supplementary Table S5). These results indicate that iridoviruses similar to CaSpA-IV are widespread and can also be detected in the environment.

### 3.4. Host DNA Exploration

To obtain some insights about the possible host of CaSpA-IV, the contigs obtained with one of the two assemblies were compared to sequences in the non-redundant NCBI database using discontinuous-megablast for taxonomy assignment. Of the 59,759 contigs with a univocal assignment (at domain level), 76.8% (N = 45,875) were classified as bacterial, while only a few could be classified as poriferal or crustacean. Specifically, 42 contigs were found that showed identity to poriferal sequences, including 29 (69.5%) mitochondrial (identity range: 78–98%) and 9 (21.4%) ribosomal (identity range: 84–99%) sequences. Additionally, as shown in Table 3, 26 sequences were identified as potentially being crustacean, with 57.7% of them (N = 15) matching sequences annotated from copepods (orders Calanoida and Siphonostomatoida). The sequences with the highest identity to reference sequences were those matching mitochondrial and ribosomal DNA of members of the order Calanoida, indicating that these zooplanktonic copepods are likely included in the diet of *C. grandis*.

## 4. Discussion

Cladorhizid sponges evolved a carnivorous feeding strategy that is unique to this family within the phylum Porifera. To be able to trap and feed on their prey, carnivorous sponges have developed specialized structures, and it was hypothesized that microorganisms play a crucial role in prey degradation and digestion since diverse sets of bacteria have been found associated with the different anatomical regions [7,8]. During a previous metagenomic investigation evaluating microbial associates in a carnivorous sponge of the species *C. grandis* we identified sequences of a novel iridovirus [12] and, in this study, we obtained and characterized the complete viral genome while investigating its association with two species of deep-sea carnivorous sponges in the Atlantic and Arctic Oceans.

### 4.1. CaSpA-IV, a Novel Member of the Iridoviridae

The combination of Illumina-based metagenomic DNA sequencing and traditional nested PCRs followed by Sanger sequencing allowed us to obtain the complete genomic sequence of CaSpA-IV. Because the iridoviral genome is terminally redundant, we inferred a circular sequence of ~190,000 bp with a GC content of 49%. This is in line with other members of the *Iridoviridae* [13]. In silico predictions identified 185 ORFs, which were found in both orientations, and approximately 77% of the resulting predicted proteins showed homology with iridoviral proteins annotated in GenBank. Among these ORFs, we could identify all 26 core genes that are usually found in all iridoviruses [13,14]. Although the genome sequence was not entirely confirmed by Sanger sequencing, the high sequencing coverage, the overall concordance of the two assemblies, and the fact that ambiguous as well as unconnected regions were verified by PCR indicate that the obtained sequence is likely correct. Nonetheless, to fully verify our findings, additional genomes should be sequenced in the future.

Genomic features, including a GC content < 50%, and phylogenetic analyses indicated that CaSpA-IV is eligible for classification as novel species within the sub-family *Betairidovirinae*. The phylogenetic tree built with a concatenated alignment of 20 core proteins showed a clear clustering of CaSpA-IV with the two known members of the genus *Decapodiridovirus* (QCIV and SHIV), which are located in an intermediate position between the *Alphairidovirinae* and other betairidoviruses. However, differently from the two fully sequenced decapodiridoviruses, CaSpA-IV possesses the gene for the small subunit of the ribonucleotide reductase. Additionally, over a concatenated alignment of 25 core proteins, CaSpA-IV was 51% and 49% identical to QCIV and SHIV, respectively, making its classification within the genus *Decapodiridovirus* uncertain. In fact, genus demarcation criteria set by the ICTV for this viral family state that “members of species within the same genus generally show greater than 50% sequence identity within a common set of 26 core genes” [13].

Interestingly, when analyzing the phylogeny of the single proteins, the three identified structural proteins of CaSpA-IV were the only ones that did not cluster within the clade of *Decapodiridovirus*. This might reflect some biological properties that differentiate CaSpA-IV from decapodiridoviruses, but further studies will be required to assess the significance of these findings. Finally, a second iridoviral lineage (CaSpA-IV-2) was identified in one *C. oxeata* specimen. Based on partial MCP sequences, this virus and a partially sequenced virus of decapods (SIV) were the closest relatives of CaSpA-IV, indicating that the three viruses may belong to the same or to closely related species. Unfortunately, the complete genome sequences of SIV and CaSpA-IV-2 will be required to define the phylogenetic relationships among the three viruses.

### 4.2. CaSpA-IV, a Virus Widespread among Deep-Sea Carnivorous Sponges

CaSpA-IV was widespread among deep-sea carnivorous sponges as it was identified both in the Gulf of Maine as well as in two locations in the Arctic Ocean (Baffin Bay) at depths of 537–852 m. Overall, CaSpA-IV was identified in eight of the nine investigated animals, and it was detected both in *C. grandis* and, at lower levels, in *C. oxeata*. These sponge species belong to two distinct, although monophyletic, genera [5,41], and they have been found to associate with different bacterial communities, resulting in seemingly host-specific microbiomes [7]. Nonetheless, it is not unprecedented to detect the same iridovirus in different, but related species as other iridoviruses were previously identified in even more distantly related hosts. For example, viruses in the species *Decapod iridescent virus 1* were found in host species belonging to different crustacean families. However, the identification of the same virus across large geographic areas reflects what was previously identified for microbial associates, which were found to be consistent across the same three locations investigated here [7].

CaSpA-IV was not consistently detected in each anatomical region of the eight positive specimens. In fact, while in animals from the Gulf of Maine, the virus was found in each tested sample, viral detection rate was lower in specimens from the Arctic. Although this could reflect some differences connected to the geographic location of the sponges or to seasonality, it could also be connected to variations during DNA isolation as the samples from the two locations were processed at different times. Overall, this virus was found in sphere, stem, and root but not in axis or embryo samples collected from *C. grandis* specimens and was detected in sphere and secondary axis but not in the stem, basal axis, or embryo samples from *C. oxeata* specimens. CaSpA-IV-2 was found only in one sphere sample collected from a *C. oxeata* specimen from Baffin Bay (Scott Inlet). This lower detectability could be connected to a lower prevalence or to an incomplete match between primers used for screening and this virus, resulting in less efficient PCRs. Nonetheless, a close inspection of CaSpA-IV reads obtained with Illumina did not show the clear presence of multiple divergent strains, at least in the Gulf of Maine. Ideally, further samples should be collected from different areas, depths, and seasons to perform a wider investigation to see if there are differences in distribution.

When we analyzed a genomic fragment characterized by the presence of a variable copy number of 10 nt long head-to-tail repeats, we found that the number of repeats was consistent within samples of the same animals but varied between animals. In two instances, the numbers of repeats were the same between samples collected in the Gulf of Maine and in the Arctic. This type of tandem repeat is common in the genome of iridoviruses, and because of the high mutation rate at the level of these sequences that cause high variation in their copy numbers, they can be used to distinguish between highly similar strains and clarify the epidemiology of iridoviruses [42]. By using this principle, we speculated that in each sponge, the same virus was predominantly present in the whole animal, while different strains were predominant in different animals. Because of the availability of a greater number of sequences for these animals, this was mainly observed for three sponges from the Gulf of Maine, in which we found viruses with identical MCP but a different copy number of repeats. This is intriguing since these specimens were recovered within 500 m from each other, and it indicates that different viral strains co-exist in the same location. The obtained results may also suggest that similar strains circulate in distant areas since sequences with the same number of repeats were found in the Gulf of Maine and in the Arctic. However, it is possible that the strains in the two locations presented additional undetected differences, so further complete genome sequencing is required to better evaluate this aspect.

### 4.3. CaSpA-IV, a Virus of Invertebrate Species X

Although CaSpA-IV is widespread in the investigated carnivorous sponges, we cannot definitively conclude that cladorhizid sponges are the (only) host for this virus. While iridoviruses can infect fish, it is likely that CaSpA-IV is a virus of invertebrates as it was found in an invertebrate that feeds on other invertebrates. Additionally, only a small difference was observed in the percentage of iridoviral reads over the total number of obtained reads when comparing the two sequencing runs made from DNA with and without methylated DNA removal (0.24 vs. 0.62%), suggesting that viral DNA was not highly methylated. This is consistent with the general observation that the DNA of invertebrate iridoviruses is poorly methylated while the one of vertebrate iridoviruses is highly methylated [13].

There are some factors that may indicate that sponges could be the actual host of CaSpA-IV. The virus was detected in carnivorous sponges of two different species and across distant geographic regions. Additionally, in some cases, the virus was found in every investigated anatomical region of one single sponge, indicating that it spread to the whole animal, and we have evidence that each sponge was colonized by one predominant strain, which differed across individuals. The obtained results are also compatible with sponges accumulating viral particles from the environment, and this could be especially true for *C. grandis* specimens since these animals do have a remnant, although reduced, aquiferous system [6]. Indeed, our investigation of public databases showed that iridoviral sequences could also be detected in the water column as well as in sediments, indicating that they can accumulate in the environment. This mechanism could explain the difference in prevalence in the two investigated areas and a reduced viral presence in *C. oxeata*, which entirely lacks an aquiferous system [6]. However, such a conclusion is poorly consistent with the uniformity of the number of repeats found in each individual.

It is also possible that CaSpA-IV infects the sponge’s prey and it becomes, during subsequent prey breakdown, distributed to the whole body of the animal. If this were the case, crustaceans seem to be the most likely culprit. First of all, crustaceans were reported as typical, although not unique, prey of carnivorous sponges [5,6], and, most importantly, CaSpA-IV is phylogenetically related to viruses found to infect decapods, with its partial MCP being approximately 97% identical to a virus found in Madagascar to be pathogenic for the decapod shrimp species *Acetes erythraeus* [16]. Our host exploration identified a very low number of contigs potentially derived from putative hosts, and this could be partially due to the fact that one of the two samples used for the metagenomic sequencing was pre-treated for methylated DNA removal, but it could also be connected to a high density of bacterial cells, which generated the vast majority of the sequences. Nonetheless, 26 contigs potentially deriving from the genetic material of four crustacean orders were identified, although contigs with high identity to reference sequences were only found for the zooplanktonic copepod order Calanoida. While not providing a conclusive answer to the host question, this at least indicates that copepods are part of the diet of *C. grandis* and that they could be a potential iridoviral host. Finally, our analyses should be repeated as more genomic sequences of deep-sea crustaceans become available, as our results may have been limited by the low number and diversity of reference sequences.

The last hypothesis is that both the sponge and its prey serve as hosts for this virus, as was documented for the lizard–cricket iridovirus (Liz–CrIV) that can infect both invertebrate and vertebrate hosts and is thought to be transmitted from insects to other hosts through predation [28]. Nonetheless, further screening studies should be carried out to identify host and transmission routes for this virus. On a technical note, the PCR we designed seems to be an ideal tool for this purpose as it can potentially detect different strains.

## 5. Conclusions

In this study, we fully genetically characterized a novel iridovirus, CaSpA-IV, that we discovered in association with the two deep-sea carnivorous sponges *C. grandis* and *C. oxeata*, from the Atlantic and Arctic Oceans. The circular viral sequence was approximately 190 Kbp in size and encompassed 185 predicted ORFs, including those coding for all 26 iridoviral core proteins. Phylogenetic analyses revealed that this virus is part of the sub-family *Betairidovirinae* and can be considered a novel species closely related to viruses within the genus *Decapodiridovirus*, while its closest relative is a partially sequenced virus of decapods identified recently in Madagascar. Screening efforts allowed us to identify the virus in several different anatomical regions of eight animals, demonstrating that CaSpA-IV is widespread over wide geographic distances and, using tandem repeat copy number analyses, we showed that the same viral strain was diffused in the whole sponge body while different but proximally located sponges contained different viral strains. While it is possible that CaSpA-IV infects the carnivorous sponges in which it was found, it is also possible that this is a virus of the sponges’ prey, possibly of crustaceans, since the virus shows close phylogenetic relationships with viruses of decapods and since genetic material of copepods was found in CaSpA-IV positive samples. However, it cannot be excluded that the virus is capable of infecting other eukaryotic hosts and of persisting freely in seawater. Given the speculative nature of some of the conclusions of this paper, further studies are required to draw more solid conclusions. Future studies should be focused on screening more sponges from a wider area as well as potential prey species and on obtaining more complete genome sequencing to elucidate the genetics, the ecology, and the evolution of this novel virus. Additionally, although challenges linked to the current impossibility of growing carnivorous sponges in laboratory settings can be foreseen, experimental studies aimed at showing whether this virus can replicate in these animals can be performed. For example, transcriptomics performed on freshly collected and properly transported material might allow the detection of viral mRNA in sponge tissues, while electron microscopy or immunofluorescence could show intracellular virus particles. Nonetheless, as more knowledge about marine invertebrates and their microbiomes accumulates, more data about the diversity and biology of deep-sea viruses will slowly surface.

## Figures and Tables

**Table 1:** Core genes and encoded proteins identified in CaSpA-IV compared to those of the reference virus (frog virus 3) and of the closest relative viruses identified with the BLASTP analysis.

Putative Function	Locus	Locus	Closest Relative to CaSpA-IV			
	FV3 ^1^	CaSpA-IV	Virus ^2^	Genus	Locus	Identity
Replication factor/DNA binding/packaging protein	1R	168R	SHIV/CQIV	*Decapodiridovirus*	2L/154R	57.0%
Serine/threonine protein kinase	57R	161R	SHIV/CQIV	*Decapodiridovirus*	12R/144L	37.0%
Myristylated membrane protein	2L	065R	SHIV/CQIV	*Decapodiridovirus*	169R/157L	37.0%
			AMIV	*Chloriridovirus*	048	37.2%
DNA polymerase family B exonuclease	60R	013R	SHIV/CQIV	*Decapodiridovirus*	110R/042L	67.5%
DNA-dependent, RNA polymerase II, α subunit	8R	131L	CQIV	*Decapodiridovirus*	097L	66.3%
DNA-dependent, RNA polymerase II, β subunit	62L	183R	SHIV/CQIV	*Decapodiridovirus*	069R/083L	67.7%
NTPase/helicase	9L	004R	SHIV/CQIV	*Decapodiridovirus*	146R/004L	57.1%
Ribonucleotide reductase, small subunit (RRα) ^3^	67L	094R	SDDV	*Megalocytivirus*	052R	58.6%
			ECIV	*Megalocytivirus*	ORF54	58.2%
Unknown function	12L	084L	SHIV/CQIV	*Decapodiridovirus*	150R/177L	38.4%
RNase III	80L	154R	SHIV/CQIV	*Decapodiridovirus*	47R/107L	57.2%
AAA-ATPase	15R	038R	SHIV/CQIV	*Decapodiridovirus*	119L/032R	72.2%
Transcription elongation factor	81R	061L	SHIV/CQIV	*Decapodiridovirus*	88R/063L	58.6%
Serine/threonine protein kinase ^4^	19R	087R	SHIV/CQIV	*Decapodiridovirus*	94L/57R	27.6%
Proliferating cell nuclear antigen ^4^	84R	002L	SHIV/CQIV	*Decapodiridovirus*	40R/114L	57.9%
Helicase ^4^	21L	173L	SHIV/CQIV	*Decapodiridovirus*	35R/120L	45.2%
Deoxynucleoside kinase ^5^	85R	083R	SHIV/CQIV	*Decapodiridovirus*	36L/118R	44.3%
D5 family NTPase involved in DNA replication	22R	039R	SHIV/CQIV	*Decapodiridovirus*	114R/037L	64.3%
Erv/Alr family of thiol oxidoreductases	88R	028R	SHIV/CQIV	*Decapodiridovirus*	-/160L	54.7%
Tyrosine kinase/LPS modifying enzyme ^4^	27R	010R	SHIV/CQIV	*Decapodiridovirus*	90R/061L	53.5%
Major capsid protein	90R	074L	SIV (partial seq.)	Unclassified	-	95.2%
			CzIV	*Iridovirus*	-	72.0%
NIF-NLI interacting factor	37R	009L	SHIV/CQIV	*Decapodiridovirus*	113L/038R	70.3%
Immediate early protein ICP-46	91R	041L	SHIV/CQIV	*Decapodiridovirus*	73R/079L	48.9%
Transcription factor	41R	170L	CQIV	*Decapodiridovirus*	145R	45.3%
			SHIV	*Decapodiridovirus*	11L	44.9%
Uvr/REP helicase	94L	093R	SHIV/CQIV	*Decapodiridovirus*	104R/048L	61.2%
Myristylated membrane protein	53R	077L	AMIV	*Chloriridovirus*	072	37.6%
			IIV25	*Chloriridovirus*	074R	37.3%
RAD2-type nuclease	95R	011R	SHIV/CQIV	*Decapodiridovirus*	31L/125R	45.8%

^1^ FV3: Frog virus 3 (accession number: AY548484). ^2^ SHIV: shrimp hemocyte iridescent virus; CQIV: Cherax quadricarinatus iridovirus; AMIV: Anopheles minimus iridovirus; SDDV: scale drop disease virus; ECIV: European chub iridovirus; SIV: sergestid iridovirus; CzIV: Costelytra zealandica iridescent virus; IV25: invertebrate iridescent virus 25. ^3^ Missing from SHIV and CQIV. ^4^ Not found in DIV-1 (Daphnia iridescent virus 1). ^5^ Missing from ECIV.

**Table 2:** Distribution of CaSpA-IV in carnivorous sponges investigated in this study.

Species	Location	Depth	Individual	Section 1	Screening	Repeats ^2^
*C. grandis*	Gulf of Maine	852 m	Mg1	Sphere	Pos	25
				Stem	Pos	25
				Root	Pos	25
		852 m	Mg2	Sphere	Pos	-
				Root	Pos	-
		852 m	Mg3	Sphere	Pos	28
				Stem	Pos	28
				Root	Pos	28
		852 m	Mg4	Sphere	Pos	17
				Stem	Pos	17
				Root (N = 2)	Pos	17
*C. grandis*	Baffin Bay (Scott Inlet)	539 m	Bg1	Sphere	Neg	-
				Stem	Neg	-
				Root tip (N = 2)	Neg	-
				Axis	Neg	-
				Embryo (N = 2)	Neg	-
		537 m	Bg2	Sphere	Neg	-
				Sphere	Pos	37
				Stem (N = 2)	Neg	-
				Axis (N = 2)	Neg	-
				Embryo	Neg	-
*C. oxeata*	Baffin Bay (Scott Inlet)	538 m	Bo2	Sphere	Pos	-
				Stem (N = 2)	Neg	-
				Secondary axis	Pos	28
				Secondary axis	Neg	-
				Basal axis (N = 2)	Neg	-
				Embryo (N = 2)	Neg	-
*C. grandis*	Baffin Bay (Davis Strait)	702 m	Bg3	Sphere (N = 2)	Neg	-
				Stem	Pos	25
				Root (N = 3)	Pos	-
				Axis	Neg	-
*C. oxeata*	Baffin Bay (Davis Strait)	680 m	Bo1	Sphere	Pos	-
				Stem	Neg	-
				Secondary axis (N = 2)	Neg	-
				Basal axis (N = 2)	Neg	-

^1^ If the number of samples investigated from a section was >1, this is indicated in parentheses. ^2^ Number of TTACGAAGAC repeats identified in the region included between nt 60,003 and 60,323; A—indicates that the PCR was negative for this sample.

**Table 3:** Contigs identified in this study that showed homology to crustacean genetic material.

N. ^1^	Best Match in the NCBI Nr Database				
	Species	Order	Region ^2^	Length (nt)	Identity (%)
5 (27)	*Calanus finmarchicus*	Calanoida	M, R	211–292	97–99
1 (2)	*Calanus hyperboreus*	Calanoida	M	211	79
3 (8)	*Eurytemora affinis*	Calanoida	G	221–225	70–75
1 (2)	*Metridia* spp./*Pleuromamma* spp.	Calanoida	R	235	97
1 (4)	*Neocalanus flemingeri*	Calanoida	R	461	81
3 (11)	*Macrobrachium nipponense*	Decapoda	G	239–309	70–87
3 (17)	*Penaeus monodon*	Decapoda	G	243–292	72–88
4 (10)	*Cyprideis torosa* ^3^	Podocopida	G	229–288	73–84
1 (7)	*Darwinula stevensoni* ^3^	Podocopida	G	109	
4 (10)	*Lepeophtheirus salmonis*	Siphonostomatoida	G	231–276	71–78

^1^ Number of identified contigs (reads). ^2^ M: mitochondrial genome; R: ribosomal DNA; G: genomic DNA other than ribosomal DNA. ^3^ Several contigs matching these species were eventually classified as bacterial.

## Data Availability

The complete genome of CaSpA-IV is available in GenBank under the accession number ON887238, while other viral sequences under accession numbers ON887239- ON887251. Crustacean reads listed in Table 3 are available as supplementary material. Illumina raw reads and alignments used for phylogenetic analyses are available upon request.

## Supplementary Materials

The following supporting information can be downloaded at: https://www.mdpi.com/article/10.3390/v14081595/s1, Table S1: Primers used in this study; Table S2: sequences used for the phylogenetic analysis of iridoviruses (Figure 3); Table S3: sequences used for the phylogenetic analysis of iridoviral major capsid proteins (Figure 4); Table S4: Overview of the shot-gun sequences; Table S5: “non-viral” contigs obtained from environmental samples that showed homology to CaSpA-IV; Figure S1: Phylogenetic analysis of proteins encoded by the core genes common to all iridoviruses; Figure S2: Partial MCP sequence alignment of CaSpA-IV and its closest relatives. Supplementary file: crustacean reads listed in Table 3.
