# Supplementary material for: A Novel Iridovirus Discovered in Deep-Sea Carnivorous Sponges
Source: Viruses. 2022 Jul 22;14(8):1595. doi: 10.3390/v14081595 (PMC9330688; doi:10.3390/v14081595)
Supplement: Supplementary file 1 [file viruses-14-01595-s001.zip › SupplementaryMaterial.pdf]

A novel iridovirus discovered in deep-sea carnivorous sponges

Marta Canuti, Gabrielle Large, Joost T.P. Verhoeven and Suzanne C. Dufour

Supplementary Material

Table S1. Primers used in this study.

Segment	Primer	Sequence	Position[1]
**MCP amplification**			
Screening	Irido_ScF	GGTAGGATTCGCTGGACTC	74,942-74,960
	Irido_ScR1	ACCTGCTCGATGAGGATGTC	74,437-74,456
	Irido_ScR2	CAGACCTGGATGTTGGTGAG	74,515-74,534
5’ sequencing	Irido_MCP-F1	CTTTACTACTGACCAATCATCC	74,389-74,410
	Irido_ScR1	ACCTGCTCGATGAGGATGTC	74,437-74,456
	Irido_ScR2	CAGACCTGGATGTTGGTGAG	74,515-74,534
3’ sequencing	Irido_MCP-F2	TCCGTAACTGGAACGAGCTG	74,622-74,641
	Irido_MCP-F3	GCTCATTCTCGACAACAGCG	74,603-74,622
	Irido_MCP-R1	CACTTAGCAGTTAGATCGATGTG	73,777-73,799
**Scaffold connecting PCRs**			
Connection A to B	IridoA_F1	CCTATCTACAGGGAGACGTC	60,003-60,022
	IridoB_R1	GTGCAAGACCAAGTGGCAAC	60,304-60,323
	IridoA_F2	TTGGGCATCCTCAGTTCACC	60,037-60,056
Connection B to C	IridoB_F1	TTCGTCGAGCTTGCGTAGC	87,104-87,122
	IridoC_R1	TCAGTGGAGGCTGTAGCTTC	88,808-88,827
	IridoB_F2	CAGCTGTTAGTTTCAAAGACATC	87,136-87,158
Connection C to D	IridoC_F1	TCTTCTATCATATCGCCCAGG	124,087-124,107
	IridoD_R1	GCCGATAGTCTTAACACCAGC	124,880-124,900
	IridoC_F2	GCGAACATCTTGACCTCCTC	124,126-124,145
Connection D to E	IridoD_F1	TGCGTGCTCACCGTTCATC	152,053-152,071
	IridoE_R1	GTCATCTGTTGAGATGCAAGG	152,754-152,774
	IridoD_F2	AAATGGAAGACCTTCGAGTCC	152,108-152,128
Connection E to A	IridoE_F1	AGTACCAGGTCTTATCGTCAG	190,027-190,047
	IridoA_R1	GCCGATGTAGGCAAACTGAAG	486-506
	IridoE_F2	ACGGGTAAGATGACCAAGGC	190,095-190,114
	IridoA_R2	CAGGTTGATGAGAACAAGACC	447-467

[1]Referred to strain CaSpA-IV MJ4 (Accession number ON887238).

Table S2. Sequences used for the phylogenetic analysis of iridoviruses (Figure 3).

Virus name	Abbreviation	Accession number
Carnivorous sponge-associated iridovirus	CaSpA-IV	ON887238
Cherax quadricarinatus iridovirus	CQIV	MF197913
Shrimp hemocyte iridescent virus	SHIV	MF599468
Invertebrate iridescent virus 31	IIV-31	HF920637
Invertebrate iridescent virus 6	IIV-6	AF303741
Lizard–cricket iridovirus	Liz-CrIV	MN081869
Daphnia iridescent virus 1	DIV-1	ERP020955
Invertebrate iridescent virus 3	IIV-3	DQ643392
Anopheles minimus iridovirus	AMIV	KF938901
Invertebrate iridescent virus 30	IIV-30	HF960234
Invertebrate iridescent virus 22	IIV-22	HF920633
Invertebrate iridescent virus 25	IIV-25	HF920635
Invertebrate iridescent virus 9	IIV-9	GQ918152
Scale drop disease virus	SDDV	KR139659
European chub iridovirus	ECIV	MK637631
Infectious spleen and kidney necrosis virus	ISKNV	AF371960
Large yellow croaker iridovirus	LYCIV	AY779031
Rock bream iridovirus	RBIV	AY532606
Orange spotted grouper iridovirus	OSGIV	AY894343
Lymphocystis disease virus 1	LCDV-1	L63545
lymphocystis disease virus China	LCDV-C	AY380826
lymphocystis disease virus Sparus aurata	LCDV-Sa	KX643370
Grouper iridovirus	GIV	AY666015
Singapore grouper iridovirus	SGIV	AY521625
Doctor fish virus	DFV	Unpublished
Guppy virus 6	GV6	Unpublished
Largemouth bass virus	LMBV	DQ159940
European catfish virus	ECV	KT989834
European sheatfish virus	ESV	JQ724856
Epizootic hematopoietic necrosis virus	EHNV	FJ433873
Short-finned eel ranavirus	SERV	KX353311
Andrias davidianus ranavirus	ADRV	KC865735
Pike-perch iridovirus	PPIV	KX574341
Frog virus 3	FV3	AY548484
Bohle iridovirus	BIV	KX185156
Common midwife toad virus	CMTVM	JQ231222
Tortoise ranavirus	TORV	KP266743
Cod iridovirus	CoIV	KX574342
Ranavirus maximus	Rmax	KX574343
Ambystoma tigrinum virus	ATV	AY150217

Table S3. Sequences used for the phylogenetic analysis of iridoviral major capsid protein (Figure 4).

Virus name	Abbre-viation	Animal in which the strain was identified					Accession number
		Name	Subphylum	Order	Environment	Area	
		**Arthropods**					
Cherax quadricarinatus iridovirus	CQIV	*Cherax quadricarinatus* (crayfish)	Crustacea	Decapoda	Freshwater	China	MF197913
		Cherax quadricarinatus (crayfish)	Crustacea	Decapoda	Freshwater	Taiwan	MW039594
Shrimp hemocyte iridescent virus	SHIV	*Litopenaeus vannamei* (prawn)	Crustacea	Decapoda	Marine	China	MF599468
		Litopenaeus vannamei (prawn)	Crustacea	Decapoda	Marine	Taiwan	MW039595
Sergestid iridovirus	SIV	*Acetes erythraeus* (prawn)	Crustacea	Decapoda	Marine	Madagascar	EF467167
Daphnia iridescent virus 1	DIV-1	*Daphnia magna* (water flea)	Crustacea	Anomopoda	Freshwater/ Brackish water	Finland	ERP020955
Invertebrate iridescent virus 9	IIV-9	*Galleria mellonella* (moth)	-	Lepidptera	Land	New Zealand	GQ918152
Invertebrate iridescent virus 25	IIV-25	*Simulium* sp. (black fly)	-	Diptera	Land	Wales	HF920635
Invertebrate iridescent virus 30	IIV-30	-	-	-	-	-	HF920636
Invertebrate iridescent virus 22	IIV-22	*Simulium* sp. (black fly)	-	Diptera	Land	Wales	HF920633
Costelytra zealandica iridescent virus	CzIV	*Costelytra zealandica* (grub)	-	Coleoptera	Land	New Zealand	AF025775
Tipoula iridescent virus type 1	TIV	*Galleria mellonella* (moth)	-	Lepidptera	Land	-	M33542
Simulium sp. iridescent virus	SsIV	*Simulium* sp. (black fly)	-	Diptera	Land	-	M32799
Invertebrate iridescent virus 3	IIV-3	*Ochlerotatus taeniorhynchus* (mosquito)	-	Diptera	Land	USA	DQ643392
Anopheles minimus iridovirus	AMIV	Anopheles minimus (mosquito)	-	Diptera	Land	China	KF938901
Invertebrate iridescent virus 31	IIV-31	*Armadillidium vulgare* (pill-bug)	Crustacea	Isopoda	Land	USA	HF920637
		Armadillidium vulgare (pill-bug)	Crustacea	Isopoda	Land	Japan	AB686457
		Burmoniscus okinawaensis (pill-bug)	Crustacea	Isopoda	Land	Japan	AB686462
		Porcellio scaber (woodlouse)	Crustacea	Isopoda	Land	Japan	AB686461
		Ligidium koreanum (woodlice)	Crustacea	Isopoda	Land	Japan	AB686458
Invertebrate iridescent virus Kaz	Kaz	-	-	-	-	Kazakhstan	MT862761
Cricket iridovirus	Cr-IV	*Gryllodes sigillatus* (cricket)	-	Orthoptera	Land	USA	OK181107
Daphnia iridescent virus 1	DIV-1	*Daphnia magna* (plankton)	Crustacea	Anomopoda	Freshwater	Finalnd	ERP020955
		**Non-arthropods**					
Carnivorous sponge-associated iridovirus	CaSpA-IV	*Chondrocladia grandis (carnivorous sponge)*	Demospongiae	Cladorhizidae	Marine	Arctic/Atlantic Ocean	ON887238
Erythrocytic necrosis virus	ENV	Chinook salmon	Actinopterygii	Salmonidae	Marine, Freshwater	Canada	MK638677

Rhinella marina erythrocytic-like virus	RMEV	*Rhinella marina* (cane toad)	Amphibia	Bufonidae	Land	French Guiana	MW582935
Lizard–cricket iridovirus	Liz-CrIV	*Trioceros hoehnelii* (chameleon)	Reptilia	Chamaeleonidae	Land	Grmany	MN081869

Table S4. Overview of the shot-gun sequencing runs.

Sample	Treatment	N reads	N reads mapped to CaSpA-IV	Percentage of mapped reads	Average coverage
Stem	Methylated DNA removed	9,492,444	22,782	0.24%	16.7X
Sphere	Untreated	7,529,438	46,669	0.62%	35X
Total	-	17,021,882	69,451	0.41%	-

Table S5. “Non-viral” contigs obtained from environmental samples that showed homology to CaSpA-IV.

**Denomination in NCBI**	**Accession number**	**Length**	**Number of proteins in the contig similar to iridoviruses**	**CaSpA-IV matching ORFs**
Nitromonadales sp.	SPBV01000186	3,986	3	168, 009, 010
	SPBV01000271	2,740	1	065
	SPBV01000285	2,580	1	004
	SPBV01000286	2579	2	069, 064
	SPBV01000293	2,470	2	012, 011
	SPBV01000295	2,432	4	016, 176, 177, 178
	SPBV01000328	2,135	2	046, 047
Pelagibacteraceae bacterium TMED237	NHKI01000013	33,658	2	0024
	NHKI01000014	24,216	6	159, 013, 021

Figure S1. Phylogenetic analysis of proteins encoded by the core genes common to all iridoviruses. The maximum likelihood phylogenetic trees were built with separate alignments of the 20 predicted protein sequences encoded by the 20 core genes listed in Table 1 that were concatenated to build the tree in Figure 3. Each tree is identified according to the gene locus in CaSpA-IV. Trees were built with IQ-Tree 2 using the best fit models identified with the ModelFinder function (168R: JTT+F+I+G4; 161R: LG+I+G4; 065R: VT+I+G4; 013R: LG+F+R4; 131L: LG+I+G4; 183R: LG+I+G4; 004R: LG+I+G4; 084L: LG+R3; 154R: LG+I+G4; 038R: LG+G4; 161L: LG+I+G4; 039R: LG+I+G4; 028R: LG+I+G4; 074L: LG+F+G4; 009L: LG+G4; 041L: LG+I+G4; 170L: WAG+F+R3; 093R: LG+I+G4; 077L: LG+F+I+G4; 011R: LG+I+G4). The outcomes of the SH-aLRT and bootstrap test (1000 replicates) are shown for the main nodes. Viruses are labelled according to their genus-level classification (*Ranavirus*: purple; *Lymphocystivirus*: red; *Megalocytivirus*: pink; *Chloridovirus*: orange; *Daphniairidovirus*: yellow; *Iridovirus*: green; *Decapodiridovirus*: turquoise; unassigned: white) and the virus identified in this study is indicated in red. Virus full names and accession numbers are available in Supplementary Table S2.

Figure S2. Partial MCP sequence alignment of CaSpA-IV and its closest relatives. Identities between sequence pairs are reported on the right.

Figure S1

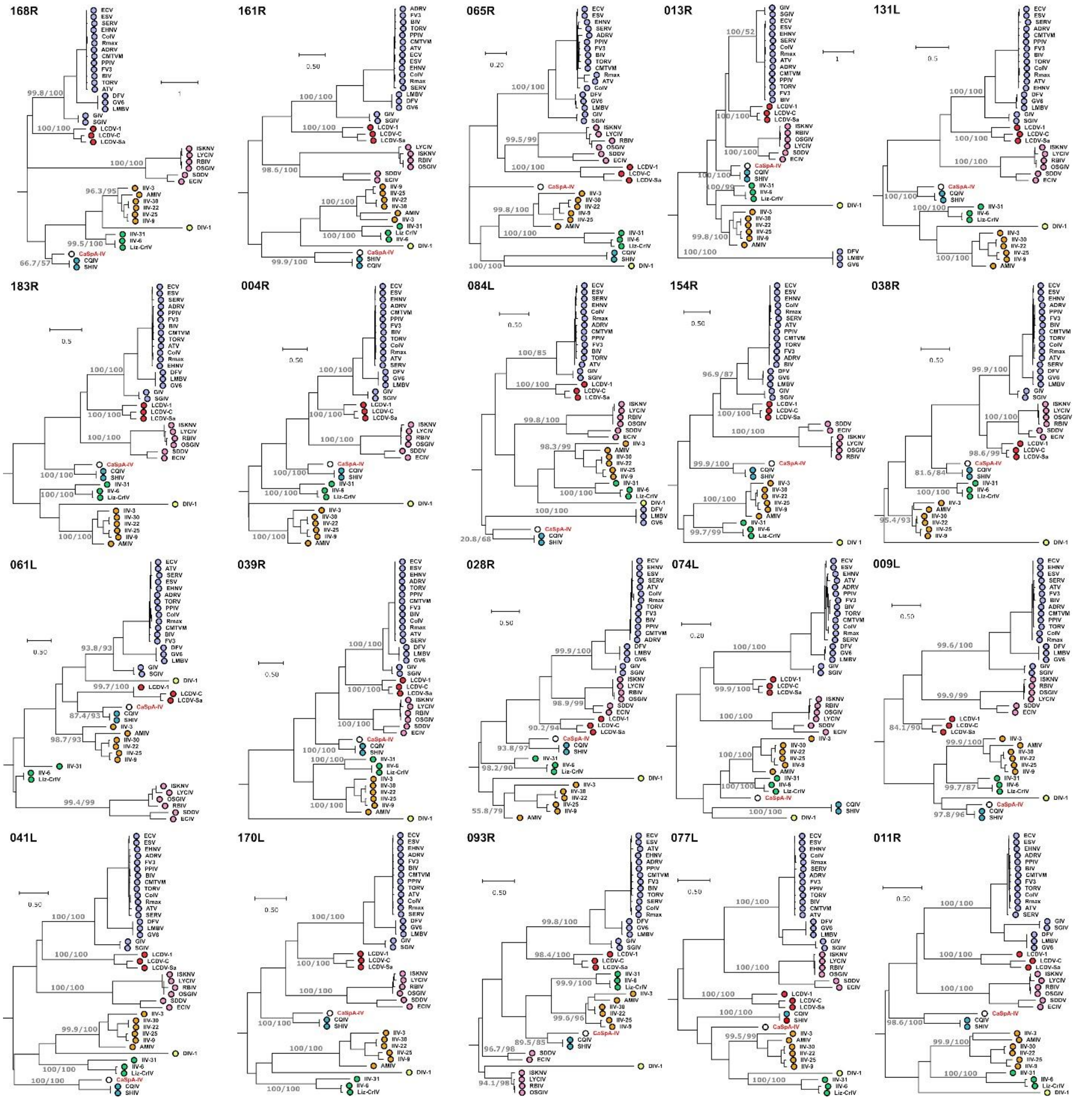

Figure S2.

```
             1        10        20        30        40        50
CASPA-IV     GRIRWTRNFMHNLIREACISFNDLVAERFDNYFLDFWSAFTVSASKRVGY
CASPA-IV-2   GRIRWTRNFMHNLIREACISFNDLVAERFDNYLLDFWSAFTVSASKRVGY
SIV          GRIRWTRNFMHNLIREACISFNDLVAERFDNYFLDFWSAFTVSASKRVGY
                      60        70        80        90       100
CASPA-IV     DNMIGNVDSLIAPHDVGVPLASQNLNLPLPFFFTRDSGVALPTAALPYNE
CASPA-IV-2   DNMIGNADSLIAPHDVGVPLASQNLNLPLPFFFTRDSGVAFPTAALPYNE
SIV          DNMIGNVDSLIAPHVVGDPLVSQNLNLPLPFFFTRDSGVALPTAALPYNE
                     110       120       130       140     148
CASPA-IV     MRISFNFRNWNELLILDNSVPVLNNNPSVVPVVGTDIAAAPELTNIQV
CASPA-IV-2   MRISFNFRNWNELLILDNSVPVLNNNPSVVPVVGTDIAAAPELTNIQV
SIV          MRISFNFRNWNELLILDNSVPVANTNPSVVPVVGTDIAAAPELTNIQV
```

	CASPA-IV	CASPA-IV-2
CASPA-IV-2	98.0%	
SIV	96.6%	94.6%
